# Sensitization for proton beam therapy using ^10^B enriched and natural isotopic abundance of boronophenylalanine

**DOI:** 10.1038/s41598-025-27619-0

**Published:** 2025-12-16

**Authors:** Shintaro Shiba, Takahiro Shimo, Masashi Yamanaka, Makoto Sakai, Tatsuya Ohno, Koichi Tokuuye, Motoko Omura

**Affiliations:** 1 Department of Radiation Oncology, Shonan Kamakura General Hospital, 1370-1, Okamoto, Kamakura, 247–8533 Kanagawa Japan; 2 Department of Radiation Oncology, Gunma University Graduate School of Medicine, 3-39-22, Showa-machi, Maebashi, Gunma Japan; 3 Radiological Research Division, Shonan Research Institute of Innovative Medicine, Shonan Kamakura General Hospital, 1370-1, Okamoto, Kamakura, 247–8533 Kanagawa Japan; 4 Department of Medical Physics, Shonan Kamakura General Hospital, 1370-1, Okamoto, Kamakura, 247–8533 Kanagawa Japan

**Keywords:** Boron, Proton beam therapy, Boron neutron capture reaction, Boron proton capture reaction, Boronophenylalanine, Cancer, Oncology

## Abstract

Nuclear reactions between proton irradiation and boron via p + ^11^B→3α (boron proton-capture reaction; BPCR) and n + ^10^B→α+^7^Li (boron neutron-capture reaction; BNCR) enhance the cell-killing effects of proton beam irradiation (PBI); however, which reaction exerts superior sensitization effects remains unclear. Therefore, we evaluated the efficacy of the BPCR and BNCR in enhancing the cell-killing effects of PBI. Human osteosarcoma (MG-63) and glioma (U-251 MG) cells were irradiated with PBI with and without boronophenylalanine (BPA) enriched with > 95% ^10^B (^10^B-BPA) for BNCR and BPA with natural isotopic abundance of boron (80% ^11^B + 20% ^10^B; N-BPA) for BPCR. Sensitizer enhancement ratios (SERs) were obtained by comparing the doses that yielded a surviving fraction of 10% (D_10_). The D_10_ values for PBI alone, PBI with ^10^B-BPA, and PBI with N-BPA were 5.67, 4.85, and 5.51 and 5.21, 4.65, and 5.18 for MG-63 and U-251 MG cells, respectively. SERs of PBI with ^10^B-BPA and N-BPA were 1.17 and 1.03 and 1.12 and 1.01 for MG-63 and U-251 MG cells, respectively. Our results indicate that ^10^B-BPA-associated BNCR may be a better sensitizer for PBI than N-BPA-associated BPCR, which warrants further investigation into the clinical use of ^10^B-enriched BPA.

## Introduction

Proton beam therapy (PBT) has been widely used as a treatment for tumors in recent years owing to its favorable therapeutic effects because of higher dose concentration properties^1–4^. However, PBT also exerts its effects on nearby healthy tissues, which hinders the increment of the radiation dose beyond the currently prescribed dose. Therefore, further improvement in the local effects of PBT may not be possible. Moreover, PBT is less effective against radioresistant tumors, because of its lower linear energy transfer (LET), than radiotherapy, which is associated with higher levels of LET, such as carbon-ion radiotherapy. Consequently, we previously conducted in vitro experiments using ^10^B-enriched boronophenylalanine (BPA) as a sensitizer and reported the sensitization effect (neutron capture-enhanced particle therapy: NCEPT) of PBT due to the boron neutron capture reaction (BNCR) caused by ^10^B and the neutrons generated in the nuclear reaction during proton beam irradiation (PBI)^5^. Generally, BNCR is more likely to occur in tumor cells expressing L-type amino acid transporter 1 (LAT1) than in healthy cells^6^. Additionally, the α particles and lithium ions generated in the BNCR have high LET radiation and are highly effective even against radioresistant tumors. Thus, NCEPT may be able to overcome the limitations of PBT, such as an increase in radiation dose, and improvement in the efficacy of PBT for radioresistant tumors.

In contrast, studies using sodium mercaptododecaborate with natural boron isotopic abundance (80% ^11^B and 20% ^10^B, N-BSH) showed a sensitizing effect of PBT via the p + ^11^B→3α (boron proton capture reaction: BPCR)^7,8^. Compared to the BNCR, the BPCR has the advantage of generating three α particles with high-LET radiations and the ability to utilize a higher particle fluence of proton particles than neutrons generated by PBI. However, the disadvantage of the BPCR is that its reaction cross section is several orders of magnitudes lower than that of the BNCR^7^.

Although the BNCR and BPCR in PBI, using a boron agent with ^10^B-enriched BPA and N-BSH, have shown increasing cell-killing effects in previous reports^5,7,8^, which reactions is a more efficient sensitizer remains unclear. Therefore, in the present study, using in vitro experiments, we aimed to evaluate the sensitizing effects of PBI using various boron agents and compare the cell-killing effects of the BNCR using ^10^B-enriched BPA (> 95% ^10^B, ^10^B-BPA) and the BPCR using BPA with natural boron isotopic abundance (N-BPA).

## Results

### Monte Carlo simulations

**Fig. 1:**
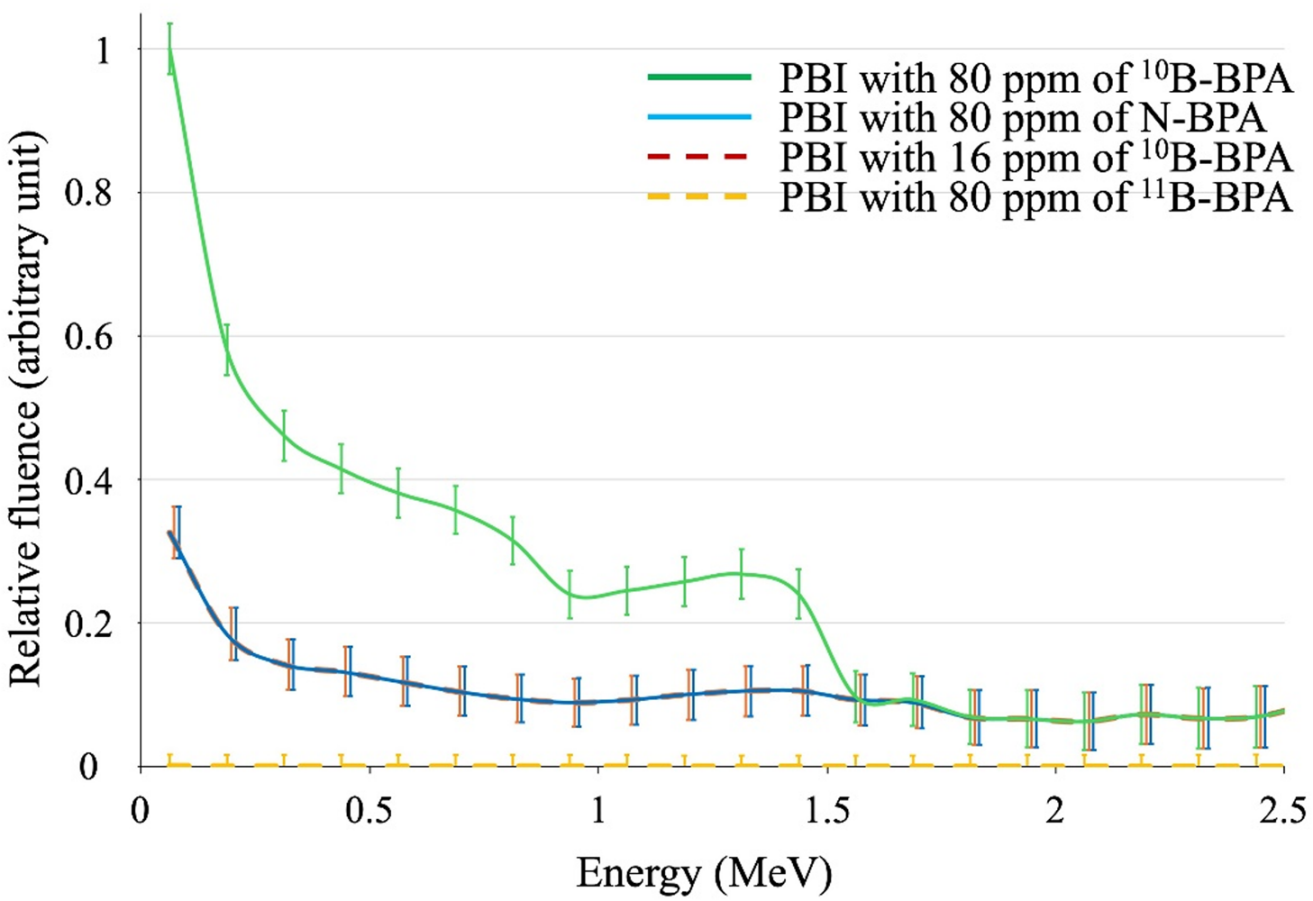
Relative total fluences of α particles and lithium ions. The relative total fluences of α particles and lithium ions increased due to PBI with 80 ppm ^10^B-BPA, PBI with 80 ppm N-BPA, PBI with 16 ppm ^10^B-BPA, and PBI with 80 ppm ^11^B-BPA administration. The statistical uncertainties of the Monte Carlo simulations are indicated by error bars. ^10^B-BPA, ^10^B-enriched boronophenylalanine; ^11^B-BPA, ^11^B-enriched boronophenylalanine; N-BPA, boronophenylalanine with natural isotopic abundance of boron; PBI, proton beam irradiation.

The relative total fluences of α particles and lithium ions generated by the administration of 80 ppm ^10^B-BPA, 80 ppm N-BPA, 16 ppm ^10^B-BPA, and 80 ppm ^11^B-BPA are presented in Fig. 1. The values compared in this simulation were obtained by subtracting BPA-free from ^10^B-BPA or ^11^B-BPA at each concentration. The total fluence ratio (sum of up to 1.5 MeV) of the α particles and lithium ions, which increased with 80 ppm ^10^B-BPA, 80 ppm N-BPA, 16 ppm ^10^B-BPA, and 80 ppm ^11^B-BPA administration, was 1.0000:0.3358:0.3356:0.0007. These results indicate that the level of α particles and lithium ions generated by ^10^B-BPA were higher than those generated by N-BPA and ^11^B-BPA.

### Intracellular Boron concentration

The mean intracellular boron concentration in the human osteosarcoma (MG-63) and human glioma (U-251 MG) cell lines was 216 (range: 199–231) and 135 ppm (range: 117–150) ppm, respectively.

### Cell-survival curve

**Fig. 2:**
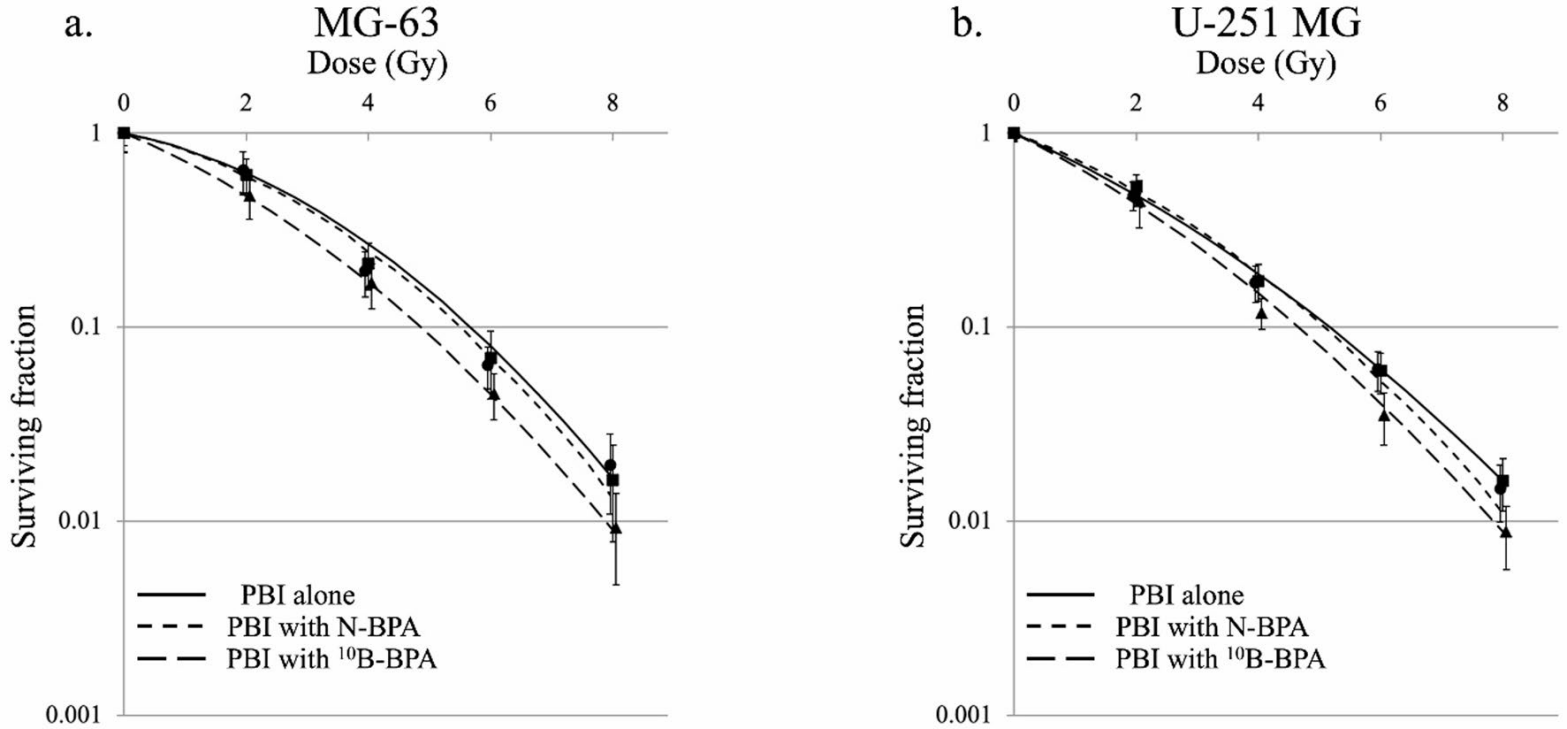
Survival curves of (**a**) MG-63 and (**b**) U-251 MG cells after the administration of PBI alone (solid line), PBI with N-BPA (dash-dotted line), and PBI with ^10^B-BPA (dashed line). The data are presented as the mean ± standard deviation, fitted to the linear-quadratic model. *P < 0.05 indicates statistical significance. ^*10*^*B-BPA* ^10^B-enriched boronophenylalanine, *MG-63* human osteosarcoma cell; N-BPA, boronophenylalanine with natural isotopic abundance of boron; PBI, proton beam irradiation; U-251 MG, human glioma cell.

The survival curves of MG-63 and U-251 MG cells under different irradiation schemes are presented in Fig. 2. The α and β values for the survival curves of PBI alone in the ^10^B-BPA and N-BPA groups were 0.145, 0.296, and 0.156, and 0.0459, 0.0366, and 0.0475, respectively, in the MG-63 cells and 0.303, 0.361, and 0.278, and 0.0266, 0.0288, and 0.0321, respectively, in the U-251 MG cells. The doses that resulted in a surviving fraction of 10% and 37% (D_10_ and D_37_) of the PBI alone in the ^10^B-BPA and N-BPA groups were 5.67, 4.85, and 5.51, and 3.33, 2.55, and 3.22, respectively, in the MG-63 cells and 5.21, 4.65, and 5.18, and 2.66, 2.32, and 2.72, respectively, in the U-251 MG cells. The sensitizer enhancement ratio (SER) based on D_10_ and D_37_ of the ^10^B-BPA and N-BPA groups was 1.17 and 1.03, and 1.31 and 1.03, respectively, in the MG-63 cells and 1.12 and 1.01, and 1.15 and 0.98, respectively, in the U-251 MG cells. The ^10^B-BPA group showed a higher SER during PBT than the N-BPA group.

Comparison of the fraction of surviving MG-63 cells under different irradiation schemes showed that PBI with ^10^B-BPA significantly reduced the cell survival compared to PBI with N-BPA and PBI alone (*P* < 0.05 and *P* < 0.05, respectively); however, no significant differences were noted between the PBI with N-BPA and PBI alone groups (*P* = 0.74). Similarly, in U-251 MG cells, PBI with ^10^B-BPA significantly reduced cell survival compared with PBI with N-BPA and PBI alone (*P* < 0.05 and *P* < 0.05, respectively); however, no significant differences were noted between the PBI with N-BPA and PBI alone groups (*P* = 0.86).

## Discussion

In the present study, we evaluated the sensitizing effect of PBI using boron agents to determine the superiority of the BNCR or the BPCR in increasing the cell-killing effect of PBI. Using in vitro experiments and Monte Carlo simulations, we confirmed that the sensitizing effects of BNCR with ^10^B-BPA on PBI were more efficient than those of BPCR with N-BPA. The α particles and lithium-ions generated by BNCR are high-LET radiation, which exhibit potent cell-killing effects. Furthermore, these particles represent ideal PBT sensitization with minimal effects on healthy cells because they selectively affect only cells that have taken up BPA. Although the sensitizing effect of the BNCR caused by ^10^B and BPCR caused by N-BSH on PBI have been previously reported, the studies separately evaluated the effect of BNCR or BPCR alone on PBI and did not directly compare the two. In contrast, we performed the first direct comparison of the sensitizing effects of the BNCR, caused by ^10^B-BPA, and the BPCR, caused by N-BPA, on PBI in vitro. Additionally, our Monte Carlo simulations showed that the nuclear reaction of PBI with ^10^B-BPA generated more α particles and lithium ions than PBI with N-BPA and ^11^B-BPA under the same boron concentrations. Consequently, ^10^B-BPA was found to be a better sensitizer for PBI than N-BPA and ^11^B-BPA. We hope that our results will encourage further investigations into the clinical use of the PBI-induced BNCR with ^10^B-BPA.

Although several studies have reported the sensitization effect of the BPCR by ^11^B, using a boron agent with N-BSH^7,8^, these studies may have involved BNCR, which is caused by 20% of ^10^B in the boron agent. However, in the current study, the boron agent in the N-BPA group contained 20% = 16 ppm ^10^B and exhibited a slight sensitization effect, which was similar to that of 20 ppm ^10^B in our previous study^5^. Additionally, our Monte Carlo simulations showed that the radiation fluences were similar for 80 ppm N-BPA and 16 ppm ^10^B-BPA. This result supports our hypothesis that the previous reports of sensitization of the BPCR using a boron agent with N-BPA were due to BNCR caused by ^10^B, which is 20% of the boron agent used in their experiments. Furthermore, a previous Monte Carlo simulation study reported that the radiation generated by the BPCR is negligible compared with that generated by the BNCR^9^. Similarly, our Monte Carlo simulations showed that the α particles and lithium-ions generated by the BNCR with ^10^B-BPA were greater than those generated by the BPCR with N-BPA and ^11^B-BPA. Thus, our in vitro experiments and Monte Carlo simulation results indicate that the ^10^B agent for the BNCR is a more efficient sensitizer for PBI than the ^11^B agent for the BPCR, under the same boron concentration.

In this study, we used MG-63 and U-251 MG cell lines, which are representative of diseases for which insufficient local control is achieved with PBT, given that this study was aimed at investigating the sensitization of PBT. However, previous studies on the BPCR have used human prostate cancer cells (DU145) and non-cancer epithelial breast cells (MCF-10 A), whereas NCEPT studies have used human glioblastoma cells (T98G), human salivary gland tumor cells (HSG), human tongue squamous cell carcinoma cells (SAS), and human malignant melanoma cells (G-361). BNCT studies using BPA involved melanoma (M8 and Mel-J) cells, those using boric acid involved human glioma cells (U-251 MG), Chinese hamster ovary cells (CHO-K1), and Chinese hamster lung fibroblasts (V79), whereas those using BPA and BSH involved Chinese hamster ovary cells (CHO)^5,7,8,10–13^. Despite these studies, additional experiments on more cell lines are required to generalize NCEPT, which is a sensitization technique for PBT using BNCR. Nevertheless, we believe that since the mechanism of boron uptake is consistent across cell lines, cells that have taken up boron in BNCT, BPCR, and NCEPT cell experiments will likely also experience the effects of NCEPT. Furthermore, a direct comparison of the cell-killing effects of BNCT alone and NCEPT is challenging because NCEPT is designed to induce a radiosensitization reaction to PBT.

Additionally, previous in vitro studies on the sensitizing effects of carbon- and helium-ion radiation by the BNCR using ^10^B-BPA showed favorable sensitizing effects^13^. Although our experimental methods and a previous report using carbon- and helium-ion beams for ^10^B-BPA administration and irradiation experimental procedures showed certain differences, both studies revealed favorable sensitizing effects of particle beams by the BNCR. This indicates that the phenomenon can be reproduced in vitro, despite the different particles, thereby supporting the future clinical use of ^10^B-BPA as a sensitizer in particle therapy. However, further studies are required to assess the clinical use of particle therapy with ^10^B-BPA, including in vivo experiments.

The results of the present study confirmed the reproducibility of intracellular boron concentration, while is consistent with our previous findings^5^. Moreover, studies have reported that transporters for BPA uptake other than LAT1, such as ATB^0,+^, become active at high BPA concentrations^6^. Notably, the boron concentration of 80 ppm utilized in this study was high, approximately 2–3 times the concentration typically employed in a clinical setting, which may have led to the activation of transporters other than LAT1, thereby contributing to increased cellular uptake. While our measured intracellular boron concentration is somewhat elevated, we contend that it is reasonable. For context, in a clinical setting, tumor concentrations are typically reported to be about 2.5 times the blood concentration^14^.

Based on our results, we believe that the tumor concentration and administration method of the boron agent must be considered before the clinical use of ^10^B-BPA as a sensitizer for PBT. The current blood concentration (i.e., extracellular boron concentration) for BNCT is approximately 30 ppm^15^. However, considering the concentration administered in the present study, it might be necessary to administer a boron agent with a concentration more than twice that currently administered in BNCT. Additionally, the dose fractionation in PBT is 10–25 fractions, which is larger than that in BNCT, and administering the drug after each irradiation can lead to physical and cost burdens. However, as particle therapy does not require as much irradiation time as BNCT, it may be possible to use a different method of administration, such as bolus administration. Therefore, this aspect should be explored in future studies.

Nonetheless, our study has certain limitations. First, this study was conducted using only two cell lines; however, we believe that the present study is sufficient to demonstrate the difference in the sensitizing effects of ^10^B-BPA and N-BPA in PBT. Second, PBI was performed using only the spot-scanning method. Given that more metal is placed on the beamline in passive-scattering PBI than that in spot-scanning PBI, which may result in higher neutron fluences, the possibility of BNCR increases. Although the present study proves that the BNCR caused by ^10^B-BPA is superior to the BPCR caused by N-BPA, further increasing the BNCR with the passive scattering method may make the BNCR more superior. Third, although the relative total radiation fluences (α particles and lithium ions) generated from the BNCR and the BPCR are given, the accurate fluence value was not calculated. As exact boron microdosimetry could not be performed in this study, future studies should aim to quantify LET and dose contributions from each nuclear reaction more precisely. Nevertheless, we believe that the Monte Carlo simulation in this study was sufficient as relatively greater α particle and lithium-ion yields were associated with a higher cell-killing effect in the in vitro experiment. However, accurate calculation and measurement of the fluences of α particles and lithium ions is an issue that should be addressed in the future.

## Conclusions

Using in vitro experiments, we demonstrated the superiority of ^10^B-BPA over N-BPA in enhancing the cell-killing effects of PBI under the same boron concentration. Additionally, our Monte Carlo simulation revealed that BNCR caused by ^10^B occurred with a higher probability and produced more α particles and lithium-ions than BPCR caused by ^11^B at the same boron concentration during PBI. This approach may open new avenues for radiosensitizer-enhanced PBT beyond the framework of conventional BNCT.

## Methods

### Cell culture

The MG-63 (Registration no.: IFO50108) and U-251 MG (Registration no.: IFO50288) cell lines were obtained from the Japanese Collection of Research Bioresources Cell Bank (JCRB). The cells were maintained in 10-cm tissue culture plates at 37 °C in a humidified atmosphere with 5% CO_2_ and Dulbecco’s modified Eagle medium containing 10% heat-inactivated fetal bovine serum and 1% penicillin-streptomycin. The medium and serum were purchased from Fujifilm Wako Pure Chemical Corporation (Tokyo, Japan). The cells were passaged before confluence and used for all experiments within 15 passages after purchase from the JCRB to obtain stable results.

### Boron compounds

BPA enriched with > 95% ^10^B (^10^B-BPA) was provided by Stella Pharma Corporation (Osaka, Japan). N-BPA (80% ^11^B and 20% ^10^B) (boron concentration, 80 ppm) was purchased from Tokyo Chemical Industry (Tokyo, Japan). ^10^B-BPA and N-BPA were added to a medium with 10% molar excess fructose (Fujifilm Wako Pure Chemical Corporation) to increase their solubility. The boron concentrations were rigorously measured using inductively coupled plasma optical emission spectroscopy (ICP-OES) (Spectrogreen, Hitachi High-Tech, Tokyo, Japan) and adjusted to a concentration of 80 ppm.

### Intracellular Boron concentration measurement

Intracellular boron concentration was measured as described previously^5^. Briefly, cells were exposed to BPA (80 ppm boron) for 2 h. Subsequently, the cell membrane was disrupted through processes such as washing and centrifugation, and the intracellular boron concentration was measured using ICP-OES three times for each sample.

### Proton beam irradiation

**Fig. 3:**
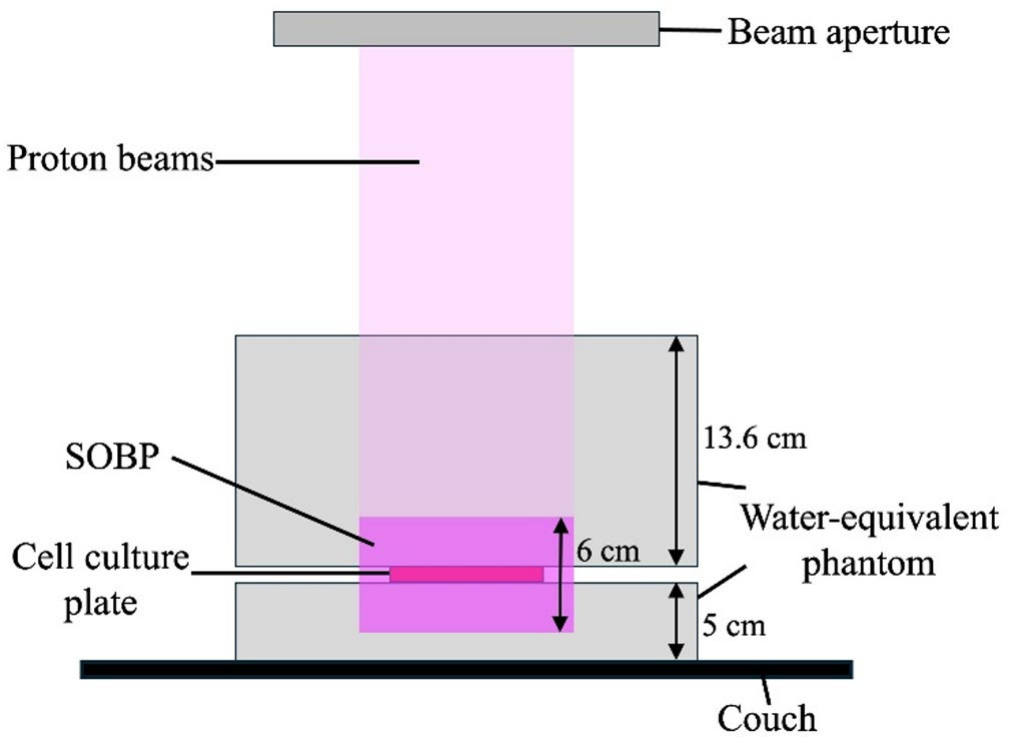
Experimental configuration of the 6-well tissue culture plate for irradiation. SOBP, spread-out Bragg peak.

PBI was performed at Shonan Kamakura General Hospital using the spot-scanning method with a spread-out Bragg peak width of 6 cm, energy range of 130.2–165.5 MeV, a spot spacing of 5 mm, and field size of 20 × 20 cm. PBI was performed using phantoms at the proximal 13.6 cm and distal 5 cm sections of a 6-well tissue culture plate, as shown in Fig. 3. The physical PBI dose was calculated using a treatment planning system (VQA, Hitachi, Japan) and the cells were irradiated at different doses (2, 4, 6, and 8 Gy). All experiments were performed in triplicates, at least.

### Experimental irradiation procedures and clonogenic cell survival assay

Cells were seeded onto 6-well tissue culture plates 24–48 h prior to irradiation. Two hours before irradiation, the cells were exposed to ^10^B-BPA and N-BPA; in the control group receiving PBI alone, only the media was changed. Subsequently, the cells were exposed (or not) to PBI. After irradiation, the BPA-containing medium in the combination groups and BPA-free medium in the control groups were discarded, and the cells were washed with PBS and suspended in BPA-free medium.

After incubation for 10–14 d, the cells were fixed in methanol and stained with crystal violet. Colonies containing at least 50 cells were counted and the surviving fractions were calculated as the ratio of surviving colonies to the number of plated cells. The surviving cell fractions were normalized to the surviving fraction in the absence of irradiation (controls) in each group (PBI alone, PBI with ^10^B-BPA group, and PBI with N-BPA group). The survival curve was approximated using the equation “S = exp (–αD – βD^2^)” with Microsoft Excel-Solver. The D_10_ and D_37_ values were calculated by substituting α and β from the approximation fitting curve into “S = –αD – βD^2^”. The SER, an indicator of the radiosensitizing effect of the PBI with ^10^B-BPA and PBI with N-BPA groups, was calculated as the ratio of cell survival (D_10_ and D_37_) after treatment with PBI alone to that after treatment with PBI with ^10^B-BPA or PBI with N-BPA.

### Monte Carlo simulations

The Particle and Heavy Ion Transport code System (version 3.31) was used for Monte Carlo simulations to calculate the fluence ratio of total radiations (α particles and lithium ions) generated from the BNCR and the BPCR^16^. The irradiation conditions for the calculations have been described previously^5^. Briefly, dose calculations were performed using the PBT system (PROBEAT-M1, Hitachi, Tokyo, Japan) at our facility under conditions of PBI using the spot-scanning method and a field size of 20 × 20 cm. The 6-well tissue culture plates were positioned at the center of the spread-out Bragg peak (6 cm wide) with an energy range of 130.2–165.5 MeV. A 30 × 30 cm water-equivalent phantom (RW3, PTW, Freiburg, Germany) was placed 13.6 cm above and 5 cm below the 6-well tissue culture plates. The cell culture plate was placed such that the center of the 1 mL medium (BPA-free, 80 ppm ^10^B-BPA, 16 ppm ^10^B-BPA, 80 ppm N-BPA, and 80 ppm of ^11^B-BPA medium) coincided with the isocenter.

### Statistical analysis

The data from independent experiments are expressed as the mean ± standard deviation. Differences were statistically analyzed using a two-sided *t*-test. Each experiment was conducted independently three times over three days. Statistical significance was set at *P* < 0.05.

## Data Availability

The datasets generated for this study are available to the corresponding author upon request.
